# Assessment and diagnosis of right ventricular failure—retrospection and future directions

**DOI:** 10.3389/fcvm.2023.1030864

**Published:** 2023-05-30

**Authors:** Sun Kyun Ro, Kei Sato, Shinichi Ijuin, Declan Sela, Gabriele Fior, Silver Heinsar, Ji Young Kim, Jonathan Chan, Hideaki Nonaka, Aaron C. W. Lin, Gianluigi Li Bassi, David G. Platts, Nchafatso G. Obonyo, Jacky Y. Suen, John F. Fraser

**Affiliations:** ^1^ Department of Thoracic and Cardiovascular Surgery, Hanyang University Guri Hospital, Hanyang University College of Medicine, Seoul, Republic of Korea; ^2^ Critical Care Research Group, The Prince Charles Hospital, University of Queensland, Brisbane, QLD, Australia; ^3^ Faculty of Medicine, University of Queensland, Brisbane, QLD, Australia; ^4^ Department of Emergency and Critical Care Medicine, Hyogo Emergency Medical Center, Kobe, Japan; ^5^ Intensive Care Unit, St. Andrews War Memorial Hospital, Brisbane, QLD, Australia; ^6^ Department of Intensive Care, North Estonia Medical Centre, Tallinn, Estonia; ^7^ Department of Nuclear Medicine, Hanyang University Guri Hospital, Hanyang University College of Medicine, Seoul, Republic of Korea; ^8^ Division of Cardiology, The Prince Charles Hospital, Brisbane, QLD, Australia; ^9^ School of Medicine, Griffith University, Gold Coast, QLD, Australia; ^10^ Division of Cardiology, Mitsui Memorial Hospital, Tokyo, Japan; ^11^ Wellcome Trust Centre for Global Health Research, Imperial College London, London, United Kingdom; ^12^ Initiative to Develop African Research Leaders (IDeAL)/KEMRI-Wellcome Trust Research Programme, Kilifi, Kenya

**Keywords:** right ventricular failure, assessment, knowledge gap, parameters, future development.

## Abstract

The right ventricle (RV) has a critical role in hemodynamics and right ventricular failure (RVF) often leads to poor clinical outcome. Despite the clinical importance of RVF, its definition and recognition currently rely on patients’ symptoms and signs, rather than on objective parameters from quantifying RV dimensions and function. A key challenge is the geometrical complexity of the RV, which often makes it difficult to assess RV function accurately. There are several assessment modalities currently utilized in the clinical settings. Each diagnostic investigation has both advantages and limitations according to its characteristics. The purpose of this review is to reflect on the current diagnostic tools, consider the potential technological advancements and propose how to improve the assessment of right ventricular failure. Advanced technique such as automatic evaluation with artificial intelligence and 3-dimensional assessment for the complex RV structure has a potential to improve RV assessment by increasing accuracy and reproducibility of the measurements. Further, noninvasive assessments for RV-pulmonary artery coupling and right and left ventricular interaction are also warranted to overcome the load-related limitations for the accurate evaluation of RV contractile function. Future studies to cross-validate the advanced technologies in various populations are required.

## 1. Introduction

Right ventricular failure (RVF) has a significant impact on the long-term outcome of various diseases, including pulmonary artery hypertension (PAH), tricuspid regurgitation, pulmonary thromboembolism, and cardiomyopathies (1, 2). However, despite its clinical importance, investigations into the right ventricle (RV) have long been neglected, with a disproportionate focus on the left ventricle (LV). Indeed, the current guidelines classify heart failure based on the LV ejection fraction (EF), without consideration of RV dysfunction (3). This could be partly due to the fact that the definition of RV dysfunction remains unclear and the choice of optimal parameters for assessing RV function is inconsistent. Additionally, it is often challenging to accurately measure the RV geometry, which has a complex morphology compared to the LV. Currently, there are several diagnostic modalities for assessing RV function, which include echocardiography, cardiac magnetic resonance (CMR), radionuclide imaging, and invasive catheter assessment. Various conventional and novel parameters have been proposed in each of these modalities, however, clinical guidelines to systematically examine patients with RV dysfunction remain less established. Hence, we focused mainly on RV dysfunction among various etiologies of RVF and aimed to review the current RV function assessment modalities by focusing on the advantages and drawbacks of each, and to suggest the future direction to improve the accuracy of RVF diagnosis.

## 2. Definition, etiology and mechanism of right ventricular failure

### 2.1. Current definitions

Heart failure is defined by the European Society of Cardiology as “a clinical syndrome consisting of cardinal symptoms (e.g., breathlessness, ankle swelling, and fatigue) that may be accompanied by signs (e.g., elevated jugular venous pressure, pulmonary crackles, and peripheral edema)” (4). It is due to structural and/or functional abnormalities of the heart which result in elevated intracardiac pressures and/or inadequate cardiac output at either rest, during exercise, or both (4, 5). More specifically, RVF has been classified as a clinical syndrome with signs and symptoms of heart failure resulting from RV dysfunction due to structural and/or functional abnormality (6). The definition of isolated RVF is less well characterized. Diagnosis is especially challenging in asymptomatic RVF which can be detected only by imaging parameters. Early diagnosis of asymptomatic RVF is often more important, which is why hemodynamic parameters obtained by echocardiography or other modalities can play an important role. However, the current classification of RVF does not include definite objective criteria referring to RV dimensions or function, but instead relies on subjective clinical symptoms. This lack of clear definitions for RVF can confound the clinical diagnosis and hence delay treatment.

### 2.2. Etiologies

Normal RV function is an interaction between preload, afterload, and contractility. Most cases of RVF occur as a result of an existing or new-onset cardiac or pulmonary disease, or a composite illness affecting both organs. RVF can result from pressure or volume overload, or decreased RV contractility, due to abnormalities of the coronary arteries, valves, pericardium, myocardium, heart rhythm, and pulmonary hypertension resulting in primary or secondary etiology (Table 1). To understand the causes of RVF, it is important to systematically assess all these functions.

**Table 1:** Etiologies of right ventricular failure.

Mechanism	Etiology	
	Acute	Chronic
Pressure overload	Pulmonary embolism	Backward LV failure (Pulmonary hypertension associated with left-sided heart disease)
	ARDS	Pulmonary valve stenosis
	Mechanical ventilation	Pulmonary artery stenosis
		Chronic thromboembolic pulmonary hypertension
		Primary pulmonary hypertension
		Chronic pulmonary disease
		Sleep-related breathing disorders
Volume overload	Excessive transfusion	Valve disease
		Pulmonary valve regurgitation
		Tricuspid valve regurgitation
		Chronic left-to-right shunt
		Atrial septal defect
		Ventricular septal defect
		PAPVR
RV contractility	RV ischemia (Myocardial infarction)	Cardiomyopathy
	Myocarditis	Arrhythmogenic RV dysplasia
	Sepsis	Amyloidosis
	Post-cardiac surgery	Sarcoidosis
	Iatrogenic/Trauma	Cardiotoxicity (Medication)

LV, left ventricular; ARDS, acute respiratory distress syndrome; PAPVR, partial anomalous pulmonary vein return; RV, right ventricular.

### 2.3. Mechanisms

#### 2.3.1. Pressure overload

Pressure overload is the predominant pathophysiologic mechanism in RVF for both cardiac and pulmonary etiologies. The concomitant occurrence of LV systolic or diastolic dysfunction and pulmonary hypertension in patients with RVF is particularly high. This corroborates the concept that the majority of RVF is secondary to left-sided cardiac disease (e.g., ischemia, mitral valve dysfunction) as post-capillary pulmonary disease or Group 2 pulmonary hypertension (7). Pressure overload is also the main etiology of RVF in patients with obstruction of the RV outflow (8).

RVF caused by pulmonary disease is well described. In 25%–60% of cases, these changes can occur suddenly, as seen, for example, in the majority of cases of massive pulmonary embolism (PE) (9, 10). An elevation in pulmonary artery pressure (PAP) following acute PE occurs when more than half of the pulmonary vasculature is obstructed by embolic material (11). In smaller embolic events, instead, the distension and recruitment of additional pulmonary capillaries can reduce vascular resistance and compensate for initial hemodynamic changes (12). An unconditioned RV can overcome a mean PAP of up to 40 mmHg in the setting of massive PE. However, further increase in RV afterload beyond this level results in RVF (13). Patients with pre-existing elevation of pulmonary pressure, instead, such as the presence of chronic PAH, can tolerate higher mean PAP > 40 mmHg acutely, due to antecedent adaptation of the RV.

Chronic lung diseases frequently affect pulmonary circulation and the right heart. Chronic obstructive pulmonary disease is the most frequent cause of respiratory failure and *Cor Pulmonale* (8). Chronic obstructive pulmonary disease increases RV afterload by several mechanisms, including hypoxia, hypercapnia, acidosis, pulmonary hyperinflation, airway resistance, and endothelial dysfunction (14). Of these factors, hypoxia is the prominent driver of pulmonary hypertension and subsequent RVF. Hypoxic pulmonary vasoconstriction results in the elevation of pulmonary pressure and, when persistent, vascular remodeling with fixed pulmonary hypertension (15).

#### 2.3.2. Volume overload

Volume overload increases RV preload due to backward LV failure (i.e., pulmonary hypertension associated with left-sided heart disease), resulting in its dilatation, which leads to augmented wall tension, and subsequently an inevitable simultaneous increase in afterload. RV interacts with the LV through the intraventricular septum. This interaction, called ventricular interdependence is defined as a direct transmission of forces affecting one ventricle to the other (16). RV dilatation or increased afterload can lead to a leftward shift of the septum, resulting in LV compression by the epicardium. Both changes result in the decrease in LV preload and contractility due to its altered geometry (e.g., pulmonary or tricuspid valve regurgitation) (17) and coronary sinus congestion, which reduces coronary flow due to elevated filling pressures on the right side of the heart and can provoke ischemia (18, 19). In patients with adult congenital heart disease such as atrial or ventricular septal defect with a clinically relevant left-to-right shunt, chronic volume overload may induce RV dilation and failure. Iatrogenic RVF through excessive transfusion is also often seen in critically ill patients.

#### 2.3.3. Right ventricular contractility

Acute reductions in RV contractility can be caused by direct myocardial injury from numerous mechanisms including myocardial ischemia and inflammation (myocarditis, sepsis) (6). Cardiac diseases involving the right heart can result in a primary reduction of RV contractility or a secondary reduction of RV preload and cardiac output, each contributing to RVF. All myocardial diseases involving the left heart may also directly or indirectly affect the RV function. These include myocardial ischemia, late-stage valvular dysfunction, dilated/hypertrophic cardiomyopathy, and cardiac amyloidosis/sarcoidosis, which is part of infiltrative cardiomyopathies Furthermore, iatrogenic RVF may also be due to some chemotherapy agents (20).

As highlighted by the heterogenous etiological and mechanistic development of RVF, a systematic approach to exclude different pathophysiological mechanisms is paramount for targeted therapy. Therefore, this review will comprehensively describe the clinical assessment of RVF.

## 3. Current assessment modalities

### 3.1. General clinical assessment

#### 3.1.1. Signs and symptoms

**Figure 1:**
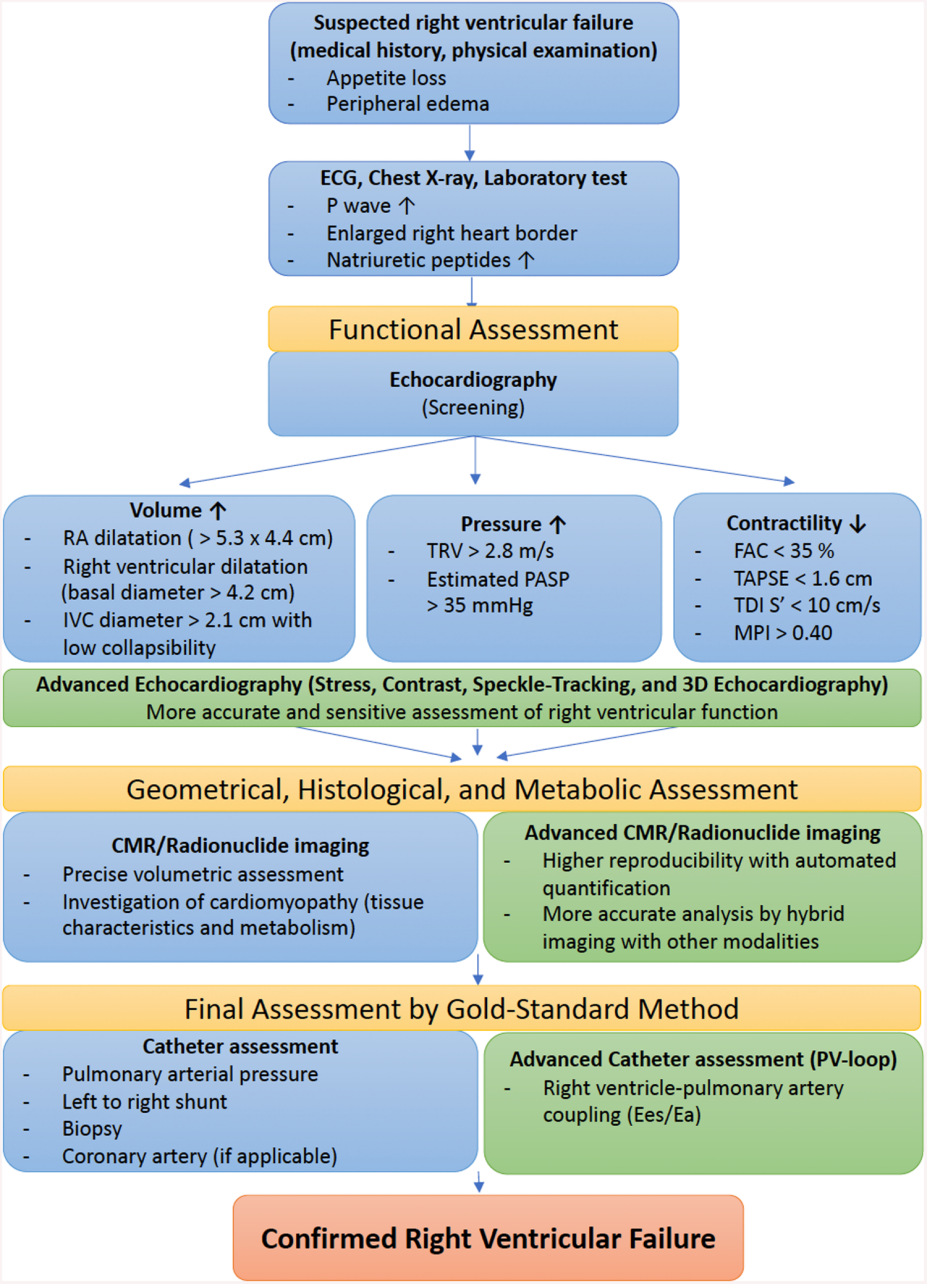
Diagnostic flowchart of basic and advanced assessment modalities for right ventricular failure. ECG, electrocardiogram; RA, right atrial; IVC, inferior vena cava; TRV, tricuspid regurgitation velocity; PASP, pulmonary arterial systolic pressure; FAC, fractional area change; TAPSE, tricuspid annular plane systolic excursion; TDI, tissue Doppler imaging; MPI, myocardial performance index; CMR, cardiac magnetic resonance; PV, pressure–volume; Ees, end-systolic elastance; Ea, arterial elastance.

Classical signs and symptoms of RVF are non-specific, as they derive from systemic venous congestion and hypoxemia (21, 22). At an early stage, patients might exhibit dyspnea on exertion, or even fatigue and rapid exhaustion and bendopnea—breathlessness when bending forward (23). As the disease progresses, however, hemoptysis, exercise-induced abdominal distension and nausea, weight gain due to fluid retention and even syncope with physical exertion may be seen. On examination, patients commonly exhibit an increased jugular venous pressure and auscultation findings such as accentuated secondary pulmonary sound (S2), RV gallop with an inspiratory pansystolic murmur over the tricuspid area (left 4th to 5th intercostal area). In addition, a prominent v wave due to increased atrial filling during systole may be seen in the central venous pressure waveform. Figure 1 illustrates a diagnostic flowchart of basic and advanced assessment modalities for RVF that will be discussed in more detail below.

#### 3.1.2. Electrocardiography

**Figure 2:**
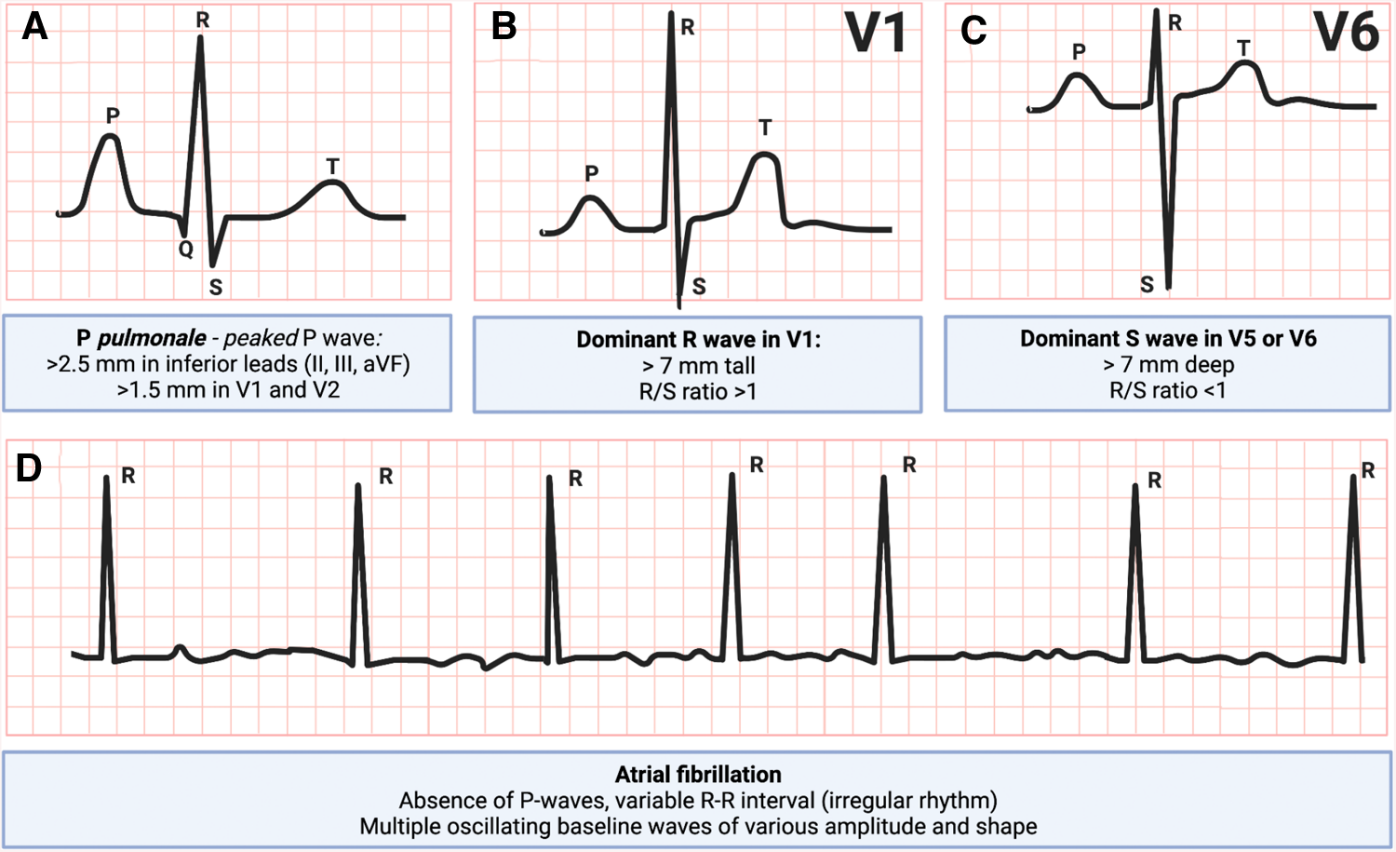
Electrocardiogram patterns commonly seen in right ventricular failure: (**A**) P pulmonale, peaked P-wave, (**B**) dominant R-wave in V1, (**C**) dominant S-wave in V5 or V6, and (**D**) atrial fibrillation (29, 30). Figure courtesy of Silver Heinsar, Critical Care Research Group, QLD, Australia.

Electrocardiography is likely the first step in workup of patients with suspected right heart issues. Patients with RV hypertrophy due to PAH have right axis deviation (axis greater than 90 degrees), features of *P pulmonale*, dominant R wave in V1 and a dominant S wave in V5 or V6 (24). In addition, atrial fibrillation is frequently encountered (25) (Figure 2). In patients presenting with an acute PE, RV dysfunction has been associated with T-wave inversions in leads V1 to V3, an S1Q3T3 pattern, and a right bundle branch block (26). Of these, T-wave inversions in leads V1 to V3 have the highest sensitivity and diagnostic accuracy for early detection of RV dysfunction in patients with an acute PE (27). Right precordial leads can assist in diagnosing RV myocardial infarction, which typically manifests as ST elevation in leads V3R and/or V4R (28).

#### 3.1.3. Chest radiography

The typical finding in right heart failure includes enlarged right heart border with cardiomegaly and distended azygous veins, yet signs of underlying causes such as pulmonary artery (PA) dilatation with distal collapse might be noted (31). Furthermore, signs of left heart failure are often observed due to its predominant etiology. In PAH or chronic thromboembolic pulmonary hypertension, enlarged pulmonary arteries with pruning of peripheral pulmonary vessels might be noted (32).

#### 3.1.4. Biomarkers

RV biomarkers can be used to assist in identifying RVF as well as monitoring disease progression. RV biomarkers can be divided to those which reflect inflammation and those which increase when myocytes are injured or experiencing stress (33). Of the latter, RVF may present with increased plasma natriuretic peptides such as N-terminal pro B-type natriuretic peptide, yet these do not delineate between left- and right-sided heart failure (34, 35). Patients who present with a RV infarct will have an increase in troponin levels (36). Additionally, cartilage intermediate layer protein 1, a novel biomarker has been associated with RV dysfunction in patients with pulmonary hypertension and ischemic cardiomyopathy (37, 38).

Of inflammatory biomarkers, suppressor of tumorgenicity 2 (ST2) and soluble ST2 (sST2) are elevated in myocyte stress, hypertrophy and fibrosis reflecting RV remodeling (39). Recent studies have implied that the levels of sST2 can reflect disease progression in patients with developing RVF (40–42). Another inflammatory molecule, Galectin 3 (Gal-3) has been shown to correlate with RV function in isolated pulmonary hypertension (43). Increased alanine aminotransferase (ALT) and aspartate aminotransferase (AST), gamma-glutamyl transpeptidase, followed by an increase in direct and indirect serum bilirubin levels can often be seen due to congestive hepatopathy (44). Lastly, markers of congestion including the neutrophil to lymphocyte ratio and AST/ALT ratio have been associated with poor prognosis (45–47).

### 3.2. Echocardiography

Among various imaging modalities for cardiac assessment, echocardiography remains the mainstay because of its availability, non-invasive nature, and cost-effectiveness (48). In particular for critically ill patients, echocardiography is available at bedside and plays an essential role to assess cardiac function. Although CMR imaging remains the gold standard of non-invasive cardiac assessment, echocardiography is comparatively more readily available and thus useful in screening for cardiac dysfunction. To assess right heart failure according to the forementioned three mechanisms, the right heart needs to be systematically assessed including the measurements of right atrial and RV chamber dimensions, RV function and right heart hemodynamics. In the following sections, we review the current best practice recommended by clinical guidelines and discuss advantages and drawbacks in each echocardiographic modality and technique. We also discuss the RV-pulmonary artery coupling and RV-LV interaction in echocardiographic RV assessment as future perspectives.

#### 3.2.1. Assessment of the right heart as recommended by clinical guidelines

Table 2 summarizes the most prevalent two-dimensional transthoracic echocardiographic parameters recommended by the American Society of Echocardiography guidelines for the assessment of right heart function (49). These key measurements with normal references are relatively simple to obtain and serve as a first step when screening for RVF. Of note, tricuspid E/A and E/E′ as a diastolic RV function have not been validated against the gold standard of end-diastolic elastance, and thus should be used with caution.

**Table 2:** Echocardiographic assessment for the right heart function recommended by guideline (49).

Parameters	Abnormal
**Right heart chamber dimension**	
Right ventricular basal diameter	>4.2 cm
Right ventricular wall thickness	>0.5 cm
RVOT diameter (parasternal short axis)	>2.7 cm
Right atrial major dimension	>5.3 cm
Right atrial minor dimension	>4.4 cm
Right atrial end-systolic area	>18 cm^2^
**Right ventricular systolic function**	
FAC	<35%
TAPSE	<1.6 cm
Pulsed tissue Doppler peak velocity at the annulus (*S*′)	<10 cm/s
Pulsed Doppler MPI (Tei index)	>0.40
**Right ventricular diastolic function**	
Tricuspid E/A	<0.8 or >2.1
Tricuspid E/E′	>6
Deceleration time	<120 ms
**Right heart hemodynamics**	
IVC diameter	>2.1 cm
IVC collapse with sniff	<50%
Tricuspid regurgitation velocity	>2.8 m/s
Systolic pulmonary artery pressure	>35 mmHg

RVOT, right ventricular outflow tract; FAC, fractional area change; TAPSE, tricuspid annular plane systolic excursion; MPI, myocardial performance index; IVC, inferior vena cava.

#### 3.2.2. Right heart assessment in intensive care

In intensive care settings, patients who are critically ill requiring urgent assessment cannot be easily mobilized. Additionally, it may also be difficult to obtain appropriate echo windows. To deal with these challenges, point of care cardiac ultrasound (POCUS) and transesophageal echocardiography (TEE) are useful tools.

##### 3.2.2.1. Point of care cardiac ultrasound

The purpose of POCUS is to obtain adequate information within the shortest time possible by applying a problem-oriented protocol for assessing specific clinical problems (e.g., cardiac tamponade) (50), and immediately determine the need for subsequent imaging (e.g., comprehensive echocardiography, computed tomography) or treatment (51). Abnormalities are typically described qualitatively (present or absent) or semi-quantitatively (normal, mild, and severe), and the assessment can be performed by non-specialists in echocardiography (50).

Right heart evaluation by POCUS includes RV size, global function, inferior vena cava (IVC) collapsibility (estimated right atrial pressure), gross tricuspid regurgitation, and the detection of pericardial effusion (50, 51). Although sensitivity and specificity of RV parameters varies among studies, those of IVC dilatation are reported as 70% and >80%, and detection of pericardial effusion is 89%–91% and 96%, respectively for RV dysfunction (52). As an example of the utility of POCUS for the right heart assessment, Taylor et al. reported that RV dilatation on emergency practitioner-performed POCUS could be an independent predictor of 30-day adverse outcomes in patients with PE (53). The application of POCUS during the COVID-19 pandemic, where rapid assessment has been required due to a lack of clinical staff and resources is another good example of the efficacy of POCUS. The American Society of Echocardiography POCUS protocol illustrates the recommended images and POCUS workflow in COVID-19 patients (51). Indeed, Zhang et al. reported the importance of POCUS in this situation to detect PE where enlarged RV and right atrium were observed and increased systolic PAP was estimated with tricuspid regurgitation and dilated IVC (53).

The major limitation of POCUS includes potential difficulties in getting appropriate echocardiographic windows due to the interruptions such as sternal wires, dressings, and chest drainage tubes. In addition, POCUS provides insufficient information for a comprehensive cardiac assessment (e.g., valvular heart disease, diastolic function, and segmental wall motion analysis) (54), and thus there is risk of overlooking important pathologic or diagnostic findings. Lack of a standardized protocols, training pathways and quality assurance also need to be resolved.

##### 3.2.2.2. Trans-esophageal echocardiography

The purpose of TEE is to obtain additional and more accurate information than transthoracic echocardiography (TTE) by taking advantage of the proximity of the esophagus to the heart (55), especially when the appropriate acoustic window is inaccessible due to patients' body habitus or intra-/post-operative conditions. TEE can assess right atrial and RV size, global right heart function, tricuspid valve regurgitation, PA size, and IVC/superior vena cava size. Although studies have suggested that measurements from TEE are almost equal to those from TTE, standard imaging planes for measurements have remained undetermined (55). TEE evaluation for the right heart may offer insights into the presence of RV infarction, pulmonary embolus, existence of pericardial effusion, and extracardiac mass effect on the RV.

One of the advantages of TEE is higher resolution images due to the proximity of the probe to the heart. In contrast, the higher frequency of the transducer reduces tissue penetration and increases attenuation, which may limit far field imaging. The effects of anesthesia on the hemodynamics can further limit assessment via TEE (55).

#### 3.2.3. Advanced echocardiographic assessment of right heart

##### 3.2.3.1. Stress echocardiography

Stress echocardiography (SE) can further assess RV function that cannot be appreciated under the rest condition by inducing stress against heart via exercise or drugs (56). SE has the potential to detect cardiac dysfunction that cannot be assessed by other modalities; (1) RV contractile reserve, and (2) stress-induced pulmonary hypertension (i.e., the early stage of PE) (57).

(1) The change of systolic parameters (tricuspid annular plane systolic excursion (TAPSE), RV fractional area change (RVFAC), S’) following stress induced can be estimated as the contractile reserve, which is the compensatory improvement of heart function during cardiac stress. Despite a lack of clear criteria of RV contractile reserve in guidelines, some revealed the prognostic significance of this value. For instance, a decline in *S*′ of >4 mm with exercise has reasonable sensitivity of proximal right coronary artery obstruction (58) Moreover, TAPSE <19 mm during exercise implies RV dysfunction that is associated with the poor prognosis in primary mitral regurgitation (59).
(2) Stress-induced pulmonary hypertension is defined as a pulmonary hypertension that happens only when stress is induced but not at rest (57). This can be also deemed as an early stage of pulmonary hypertension. SE is essential to diagnose this stress-induced pulmonary hypertension and the result can be a better prognostic value in this cohort. For example, the estimated systolic PAP ≧60 mmHg during SE has been reported as a predictor for poor clinical outcomes (60).

The disadvantages of SE include a risk of deterioration in heart failure and arrhythmias (61), thus it cannot be conducted in critically ill patients such as those with acute coronary syndromes, severe cardiac arrhythmias, and acute heart failure.

##### 3.2.3.2. Contrast echocardiography

Contrast echocardiography (CE) is indicated to improve structural or functional assessment of cardiac chambers and masses under sub-optimal image quality (62). Besides, CE can also be used to visualize RV perfusion, and to identify the right-to-left shunts.

CE can clearly visualize the RV endocardial border and allow more accurate assessment of RV dimensions and regional wall motion. By combining CE with 3-dimensional (3D) echocardiography, more accurate and reproducible measurements can be obtained (63). In cases with coronary artery disease or RV cardiomyopathy, myocardial CE may be able to visualize RV perfusion (64, 65) that cannot be detected by other echocardiographic techniques.

Limitations include workflow issues (such as the need for intravenous cannulation), potential artifacts, and lack of standardized normal values of chamber volumes (48).

##### 3.2.3.3. Speckle-tracking echocardiography

Speckle-tracking echocardiography (STE) is one of the advanced analyses to assess cardiac function with higher precision than previous echocardiographic parameters (66, 67). Cardiac chamber deformation can be measured by speckle tracking technique as strain (%), which is more sensitive and thus can detect subtle dysfunction that cannot be revealed by conventional echocardiographic parameters including RVFAC, TAPSE and S’. Besides, STE could be less load dependent than other conventional parameters by focusing on myocardium itself rather than volume change in the cardiac evaluation.

RV longitudinal strain has been mostly used for the assessment in the RV contractility among several STE parameters. In addition, there are two methods to assess RV longitudinal strain (RVLS); one is RV free wall longitudinal strain (RV-fwLS) and the other is RV global longitudinal strain (RV-GLS). RV-fwLS is based on the evaluation of only free wall of RV, while RV-GLS additionally includes intraventricular septum area. The normal values differ between the two parameters (RV-fwLS < −20% and RV-GLS < −14%). The American Society of Echocardiography/European Association of Cardiovascular Imaging/Industry Task Force recommends reporting RV-fwLS, while RV-GLS is optional (68).

RVLS has two notable characteristics; (1) the improved accuracy enough to identify an early RV dysfunction, and (2) a regional assessment of RV Function.

(1) RVLS can reveal an early-stage RV dysfunction and can be useful as a better prognostic predictor (67, 69–72). For instance, RV-fwLS has been demonstrated as a better predictor than TAPSE for PAH-related clinical events (hospitalization and therapeutic intervention) and all-cause mortality during 2.0–3.8 years follow up in a mixed etiology of PAH (73, 74). Moreover, improvement of RV-fwLS following treatment in PAH was associated with a lower mortality, suggesting its possibility as a therapy target (75).
(2) With the advantage of being angle independent unlike the Doppler technique, STE analysis provides not only global but also regional assessment that could be helpful for differentiating the etiology of diseases such as atrial septal defect, arrhythmogenic RV cardiomyopathy and cardiac amyloidosis (76–79).

##### 3.2.3.4. Three-dimensional echocardiography

Three-dimensional (3D) echocardiography is helpful to accurately measure RVEF, which is one of the most reliable RV contractile parameters (80). It is difficult for two-dimensional (2D) images to accurately evaluate RVEF because the RV has a crescent shape around the LV, and thus its whole image cannot be simultaneously captured by using the conventional 2D imaging technique. By using 3D echocardiography, RVEF can be measured in congenital heart diseases where patients have unique RV morphology. RVEF derived from 3D echocardiography has been shown to significantly correlate with CMR-derived measurement, widely considered as the gold standard assessment of RVEF (81–83). The limitations of 3D echocardiography are lower resolution, unstandardized normal values, and high dependence on image quality (48).

#### 3.2.4. Future perspectives in echocardiography

Although RV function and size are significantly affected by PAP as well as LV function, vast majority of echocardiographic parameters of RV contractility recommended by the American Society of Echocardiography guidelines (49) and European Society of Cardiology (84, 85) do not consider the inter-relationship between RV function and PAP or LV function. Therefore, the concepts of the RV-PA coupling and RV-LV interaction in echocardiographic assessment are crucial to comprehensively describe RV function.

One of the parameters considering the inter-relationship between ventricular function and arterial pressure is stroke work (SW). Conventionally, SW was described as the area surrounded by the catheter-based pressure-volume (PV) curve. However, Russell et al. reported that myocardial work (MW), a non-invasively measured STE parameter, is well corelated with invasively measured LV SW (86). The clinical utility of LV MW is supported by other studies, where MW can provide better understanding of the LV remodeling under different loading conditions, and also predict significant coronary artery disease in patients with normal LV function and wall motion (87, 88). Investigation into whether MW-based STE can be applicable in RV assessment is warranted.

The gold standard of RV-PA coupling is described as the ratio of end-systolic/arterial elastance (Ees/Ea).This value plays a key role for the detection of RV dysfunction in the early stage of PAH (66) because Ees/Ea is a sensitive marker of RV dysfunction, which starts to decrease before EF deterioration or RV dilatation are observed (89). This may facilitate recognition of adaptive (compensated) and mal-adaptive (decompensated) phases of RVF interchange (67, 89), and thus avoid delays in commencing treatment.

Ees/Ea is derived from an invasive assessment using the PV loop (as described below) and thus has not been commonly used in both clinical practice and research since its introduction a decade ago. Therefore, a less-invasive surrogate maker of Ees/Ea is earnestly desired. Tello et al. reported on the utility of TAPSE/systolic PAP as a surrogate of Ees/Ea with simplified methodology by using 2D echocardiography and found that this value can be a predictor of poor outcomes in PAH (90). Also, Richter et al. reported that the 2D and 3D echocardiography derived Ees/Ea (i.e., SV/ESV) could well correlate with the invasively measured Ees/Ea (91). However, in order to utilize these non-invasive values in clinical settings, further validation studies are required.

### 3.3. Cardiac magnetic resonance

CMR is the gold standard non-invasive diagnostic tool for assessment of cardiac structure and function. It can provide static images using the spin-echo sequence as well as multiple images by using the gradient-echo sequence during the cardiac cycle (92). It acquires full biventricular images by 3-dimensionally piling contiguous short-axis cines to measure ventricular volume and mass. Biventricular volumes and mass are determined by planimetry for each slice and summed for the whole ventricle (92). Any desired imaging planes can be safely provided without administration of contrast agents or exposure to ionizing radiation.

Current guidelines for heart failure still recommend TTE as an initial imaging modality; however, they also recommend CMR to assess myocardial structure and function in patients with poor echocardiogram acoustic window and to characterize the myocardial tissue in suspected infiltrative and inflammatory diseases including myocarditis, sarcoidosis, Fabry disease, Chagas disease, noncompaction, iron overload, amyloidosis, and cardiomyopathies (Table 3) (3, 4). In particular, non-contrast T1 and T2 images and nonischemic patterns of late gadolinium enhancement can yield more information for many cardiomyopathies (93–96).

**Table 3:** Current guidelines for using cardiac magnetic resonance in evaluation of heart failure (3, 4).

Recommendations	Guidelines	COR	LOE
In patients for whom echocardiography is inadequate	2022 AHA	I	C
	2021 ESC	I	C
For the characterization of myocardial tissue in suspected infiltrative diseases	2021 ESC	I	C
In patients with heart failure or cardiomyopathy	2022 AHA	IIa	B
In patients with heart failure, an evaluation for ischemic heart diseases	2022 AHA	IIa	B
In patients with dilated cardiomyopathy to distinguish between ischemic and nonischemic myocardial damages	2021 ESC	IIa	C
In patients with heart failure and coronary artery disease to assess myocardial ischemia and viability for coronary revascularization	2022 AHA	IIb	B
	2021 ESC	IIb	B

AHA, American heart association; ESC, European society of cardiology.

#### 3.3.1. Advantages

CMR can provide accurate and reproducible measurements of the LV parameters (97–99). According to the interstudy comparison, CMR showed superior reproducibility for assessment of LV volumes, mass, and EF over 2D echocardiography (98). In this aspect, CMR is also suitable for the assessment of the RV, despite its variable configuration that it is often challenging to define geometrically. Indeed, the accuracy of CMR for measurement of RV parameters in various disease has been corroborated by several studies (101–103). Grothuses et al. reported that the reproducibility of RV measurement with CMR was optimal in patients with LV hypertrophy and heart failure as well as in healthy individuals (104). With increasing availability of CMR in the clinical settings, its application techniques have been continuously improved. The steady-state free precision yields significantly improved blood-myocardium contrast, acquisition speed, and the ability to greatly improve the temporal resolution of the cines with improved image quality (105). This technical improvement eventually has led to the adjustment of RV measurement for age, gender, and body surface area, which is important to detect smaller changes in earlier stage of disease (106). Quantification of myocardial strain can also be performed using CMR feature tracking. Indeed, Lin et al. conducted RV strain analysis during exercise using CMR and reported that RVLS was significantly more sensitive than RVEF in detecting early RV systolic dysfunction in PAH (107).

#### 3.3.2. Disadvantages

CMR is expensive and its access can be limited in some countries and centers, although some authors reported that CMR could reduce the cost of surveillance in patients with RVF (108). They concluded that lower measurement variability has led to the allowance of smaller sample size in PAH drug trials as well as reduced overall costs. Availability and access issues can limit CMR as a standard longitudinal assessment modality for patients with RVF. In particular, its accessibility to critically unstable patients may be very limited, whereas TTE can be easily performed at the bedside. Its utilization could also be restrictive in patients with cardiac implantable electronic cardiac devices, the requirements of which is steadily increasing (109). CMR may cause malfunction of the implanted devices such as an implantable cardioverter defibrillator and other pacemakers and these devices induce metal artifacts to hinder the diagnostic image quality. The MR-conditional pacemaker system has been developed and a protocol-based practice to prevent adverse events can be applied for safety (110). An inversion recovery technique to alleviate the metal artifacts without increasing scan time has also been developed (111).

#### 3.3.3. Future perspectives in cardiac magnetic resonance

Recently, artificial intelligence (AI)-based fully automated analyses were introduced to overcome the observer-variability resulting from manual preparation and reselection of CMR images (112). According to one recent study by Backhaus et al., however, manual post-processing correction by an experienced observer is still necessary for RV assessments because fully automated analyses do not provide satisfactory reproducibility (99). Further development of deep learning techniques to maximize the reproducibility by minimizing manual manipulation is anticipated.

Assessment of RV contractile reserve in PAH patients under stress conditions allows early detection of subclinical disease. Recently introduced CMR, using ultra-fast compressed sense sequence, exercise CMR allows accurate assessment of cardiac function under stress conditions in PAH patients. Lin et al. examined effects of exercise on the RV and demonstrated significantly higher RV contractile reserve in controls compared to PAH patients and the RV-PA coupling ratio failed to increase in PAH patients during exercise (113).

### 3.4. Radionuclide imaging

Approximately 50 years ago, Strauss and his colleagues reported recognizable nuclear cardiac images which could measure LVEF in patients without cardiac catheterization by using a method they referred to as “gating” (114). Ever since, radionuclide ventriculography (RVG) with first-pass and equilibrium techniques has been widely utilized in cardiac imaging. Gated equilibrium RVG is performed through electrocardiography gating. Alternative terminologies for this technique include gated cardiac blood pool imaging, multigated acquisition, and gated equilibrium radionuclide angiography (115). The majority of these studies are performed for assessment of LV function including EF, intraventricular volumes, and ventricular wall motion (116). Furthermore, radionucleotides can be used for assessment of RV function before LVAD implantation as RV heart failure is a common and major complication of this procedure (115, 117). Radionuclide imaging has stood the test of time as it has proven to be an excellent noninvasive modality with high reproducibility, lower inter- and intraobserver variability compared with 2D echocardiography (118). Several previous studies reported that gated blood pool single photon electron computed tomography showed similar accuracy for the estimation of the RV volume and function to CMR and low inter-observer variability (117, 119). In addition, RVG can be used in patients with CMR-incompatible implanted electronic devices. However, its limitations are radiation exposure due to radiopharmaceutical injection, occasional poor image quality due to inadequate labeling and the chance of inaccurate gating due to arrhythmias (e.g., frequent premature ventricular contractions and atrial fibrillation) (120).

Chronic RV pressure or volume overload are known to change the myocardial energy preference (121–123). Chronic ventricular conditions including an atrial septal defect or PAH can change myocardial metabolic function. [^18^F] fluorodeoxyglucose positron emission tomography can demonstrate changes in myocardial glucose metabolism in these conditions (124–126). [^18^F] fluorodeoxyglucose accumulation in the RV free wall significantly increased in accordance with the severity of the RV pressure overload (124) and was a poor prognostic indicator for long-term outcomes in patients with PAH (125). Myocardial glucose utilization in the interventricular septum relative to the LV free wall increases in relation to long-term RV volume overload in patients with an atrial septal defect (126).

#### 3.4.1. Future perspective in radionuclide imaging

Image quality and contrast resolution of radionuclide imaging of the heart have continuously improved due to several technical advances such as single photon emission computed tomography, positron emission tomography, and hybrid single photon emission computed tomography/computed tomography and positron emission tomography/computed tomography systems, which can show physiologic and anatomical information of the RV simultaneously. These improvements in radionuclide cardiac imaging will provide new opportunities for more accurate assessment of the function of RV as well as RV myocardial metabolism in the near future.

### 3.5. Invasive pressure-volume analysis

**Figure 3:**
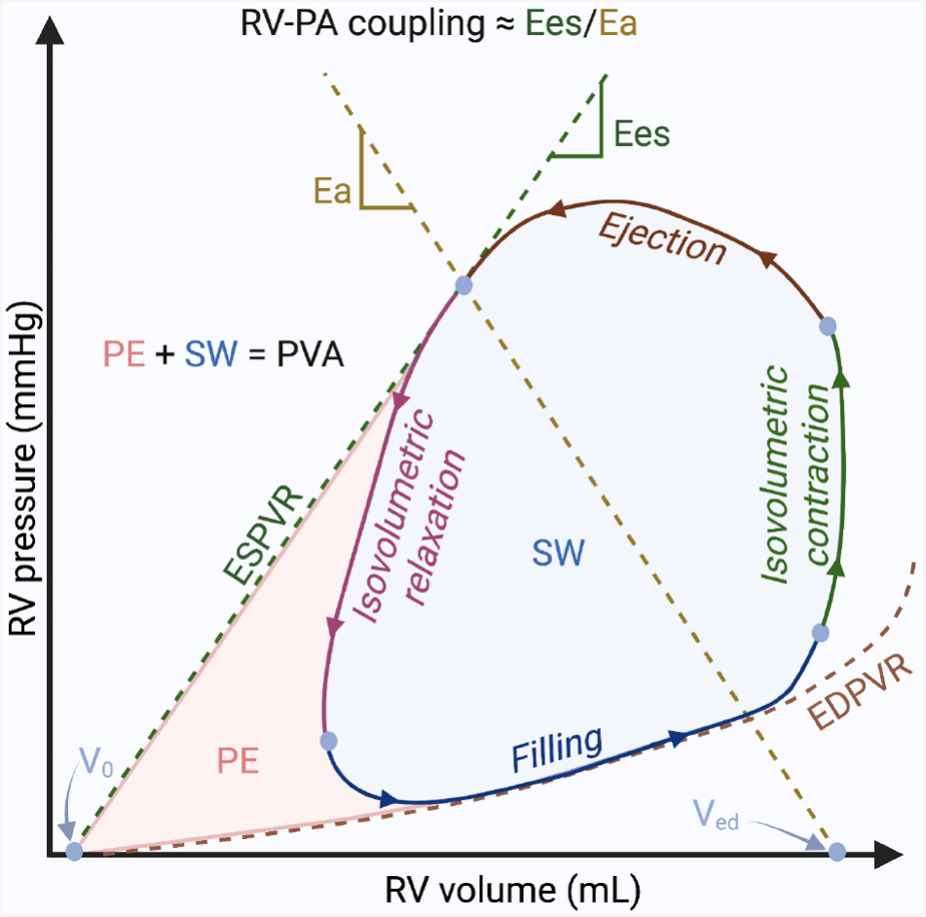
Basic elements of the right ventricular pressure-volume loop diagram. The diagram illustrates the relationship between the pressure and the volume in the right ventricle during the 4 phases of one cardiac cycle: ventricular filling, isovolumetric contraction, ejection, and isovolumetric relaxation. Pressure–volume (PV) relationships. Connecting the end-systolic PV coordinate with the unstressed blood volume of the ventricle (volume-axis intercept—*V*_0_), the end-systolic PV relationship (ESPVR) describes the load-independent cardiac contractility; the end-diastolic PV relationship (EDPVR) describes the load-independent ventricular diastolic function. Myocardial energetics. The sum between the stroke work (SW) described by the area within the loop and the potential space bounded within the ESPVR and EDPVR outside the loop (potential energy—PE) is called the PV area (PVA), which is linearly related to myocardial oxygen consumption. Right ventricle (RV)-pulmonary arterial (PA) coupling. The pulmonary arterial elastance (Ea) is described by the connection between the end-systolic PV coordinate with the *V*_0_ at end-diastolic volume (*V*_ed_). The end-systolic ventricular elastance (Ees), is described by the slope of ESPVR. The ratio between Ea and Ees (Ea/Ees) is currently the gold-standard to describe the RV-PA coupling. Figure courtesy of Silver Heinsar, Critical Care Research Group, QLD, Australia.

PV analysis is considered the gold-standard method for characterizing ventricular systolic and diastolic function, as well as ventricular-vascular interactions (127). However, the complexity and invasiveness of this technique have prevented its routine use in clinical practice, limiting its current application to a few particular cases or for research purposes. By simultaneously plotting real-time ventricular pressure against volume during a cardiac cycle (or heartbeat), PV loops provide a unique, quantitative approach for determining the load-independent (not influenced by the preload or afterload of the heart) contractility of the heart. PV loop analysis is performed with a specialized high-fidelity conductance catheter, inserted from either the right internal jugular or common femoral vein, and positioned into the RV apex. The catheter features a solid-state pressure sensor in the middle of an array of equally spaced electrodes. Segmental PV loops are produced from each adjacent pair of electrodes, and these segmental volumes are summated to produce the overall ventricular volume. While the normal LV PV loop has a square shape with end-systolic pressure easily identified at its upper left corner, the normal RV PV loop has a rounded shape with early systolic peaking of pressure (128). Following the wide application to understanding LV mechanics in health and disease, this technique has also recently gained a lot of interest in the assessment of the RV function. Figure 3 shows the basic elements of the RV PV loop diagram.

#### 3.5.1. Conductance catheter: advantages and drawbacks

Like echocardiography and CMR, PV catheters can measure both end-diastolic and end-systolic volumes, and from these, calculate the stroke volume, EF, and cardiac output. Additional parameters, such as SW, isovolumetric index, contraction time, and relaxation time, can also be calculated. However, these parameters are all considered to be load-dependent. True contractility is a load-independent phenomenon, reflecting the innate ability of the myocardium to exert force, and measuring load-independent parameters is one of the main advantages of PV loop analysis. Indeed, describing ventricular systolic and diastolic function is among the key objectives of PV analysis, and it can be achieved by characterizing the end-systolic and end-diastolic PV relationships, which are load-independent measures of cardiac contractility and compliance, respectively. Two different techniques have been described to obtain these relationships using the PV catheter: the multi-beat and the single-beat methods (128, 129). Both techniques have been validated for the RV PV loop (130–132), each with its pros and cons; two detailed reviews on these methods should be used for greater detail (133, 134). The slope of end-systolic PV relationship (which represents the maximal pressure generated by the ventricle at any given volume) is also referred to as Ees and is considered the gold standard for measurement of RV contractility (135). In addition, because of the major prognostic relevance of RV function in the context of patients with PAH (2), the characterization of the relationship between RV and PA (RV-PA coupling) has recently gained a lot of interest. To assess the adequacy of RV contractile adaptation to afterload, Ees is expressed in relation to Ea, defined as the relationship between end-systolic pressure and stroke volume (2, 135). The optimal coupling of the RV to afterload allowing for flow output at a minimal energy cost occurs at an Ees/Ea ratio of around 1.5 (135). An earlier and robust diagnosis of RV-PA uncoupling and definition of critical Ees and Ees/Ea values associated with a risk for clinical deterioration may have crucial importance in patients with PAH (136, 137).

The main disadvantage of using a conductance catheter is that the technique is inherently invasive. Catheter positioning and other manipulations should be performed under fluoroscopic guidance, and studies in patients with a recently implanted pacemaker or defibrillator lead should be avoided. The conductance catheter must be occasionally retracted off the RV wall to quell arrhythmias. At times, the conductance catheter may tether the tricuspid valve and introduce tricuspid regurgitation, especially when additional catheters (i.e., Swan-Ganz catheter) are also crossing the valve. Furthermore, the calibration technique is challenging, and, very importantly, a standardization of interpretation of the loops is still needed as the technique becomes more widely adopted and incorporated into various research and clinical practices (134).

#### 3.5.2. Clinical applications

With improved catheter technology and easier signal processing, RV PV loop analysis is being performed with increased regularity in several clinical scenarios (134). As discussed, patients typically undergo non-invasive evaluations of RV function with multiple different modalities. PV analysis can help in resolving the remaining uncertainties by quantifying RV systolic and diastolic function and the nature of RV-PA coupling. Current applications of PV loop analysis in the clinical scenarios include:

(1) PAH: PV analysis can identify occult RV dysfunction (RV-PA coupling assessed by Ees/Ea ratio) with greater sensitivity than traditional measurements (138, 139) and better predict clinical worsening (140).
(2) Heart failure with preserved EF: using the end-diastolic PV relationship, PV analysis can reveal impairments in diastolic function (such as increased load-independent RV stiffness and impaired active relaxation) (141). Considering the prevalence of heart failure with preserved EF and the many unanswered questions regarding the pathophysiology of this condition, PV analysis is primed to play an important role in future studies (134).
(3) End-stage heart failure supported by LVAD: PV analysis in LVAD recipients can demonstrate that even patients with echocardiographically normal appearing RV have only modest inotropic reserve during exercise and experience dramatic increase in LV filling pressures (142). The ongoing REVIVAL-VAD trial (NCT01369407) will be an important proof-of-concept study for using PV analysis in this clinical context.
(4) Valvular Heart Diseases: the broad variability in outcomes seen in real-world practice following technically successful transcatheter and surgical aortic valve replacement may be partially explained by pre-procedural and procedural RV dysfunction (143). In addition, given the strong interaction between mitral regurgitation or tricuspid regurgitation and RV function, atrioventricular valve diseases may also benefit from a periprocedural RV function assessment (144). Both these clinical scenarios present very interesting opportunities to evaluate RV physiology with PV analysis (134).

#### 3.5.3. Future perspectives in pressure–volume analysis

PV loops are an accurate means of recognizing the uncoupling of the ratio of PA to RV end-systolic elastance characteristic of RVF (145). However, their application in clinical practice is limited due to the level of invasiveness in PV loop acquisition (67, 89). Thus, reconstructing PV loops using non-invasively obtained data is becoming increasingly relevant. To this end, CMR and echocardiography have been used to reconstruct elements of the PV loop and estimate RV-PA coupling (90, 146, 147). The utility of CMR however, is limited by cost and availability, as well as its reliance on patient cooperation, a significant barrier in the critically ill. Echocardiographic reconstruction of RV PV loops is achieved by synchronizing volume-time curves (generated with 3D echocardiography) and pressure-time curves (generated by measuring RV pressure either with an intraventricular wire or with echocardiography aligned with specific events during the cardiac cycle) (91). Another promising non-invasive PV loop reconstruction comes from the latest development in the evolution of strain imaging. Indeed, a novel method for assessing LV work using pressure-strain loops during echocardiography has been proposed as mentioned in the section of “Future perspective in echocardiography”. The pressure-strain loops are created by combining a surrogate of LV pressure, noninvasively estimated using systolic blood pressure synchronized and normalized with echocardiography-derived valvular timing events, with longitudinal strain, measured using standard 2D STE (148). The area of this combined non-invasive LV pressure-strain loops has been validated to correlate with invasive myocardial work and reflects glucose metabolism by [^18^F] fluorodeoxyglucose positron emission tomography (149). This new type of echocardiographic index may offer a more detailed assessment of intrinsic myocardial function by incorporating both strain and afterload simultaneously, adding incremental value to existing strain evaluation (148). Currently used only for LV assessment, time will tell whether myocardial work derived with this innovative technique will eventually play a successful role in RV assessment as well.

There are still many limitations in these non-invasive methods, but their potential is significant: by easing the barriers to data acquisition, non-invasive PV loops may become a fast, convenient way to monitor changes in ventricular function over time or in response to certain interventions (134). In order to utilize these non-invasive values in clinical setting, further validation studies are required.

## 4. Novel future technologies of right ventricular failure assessment being developed

**Figure 4:**
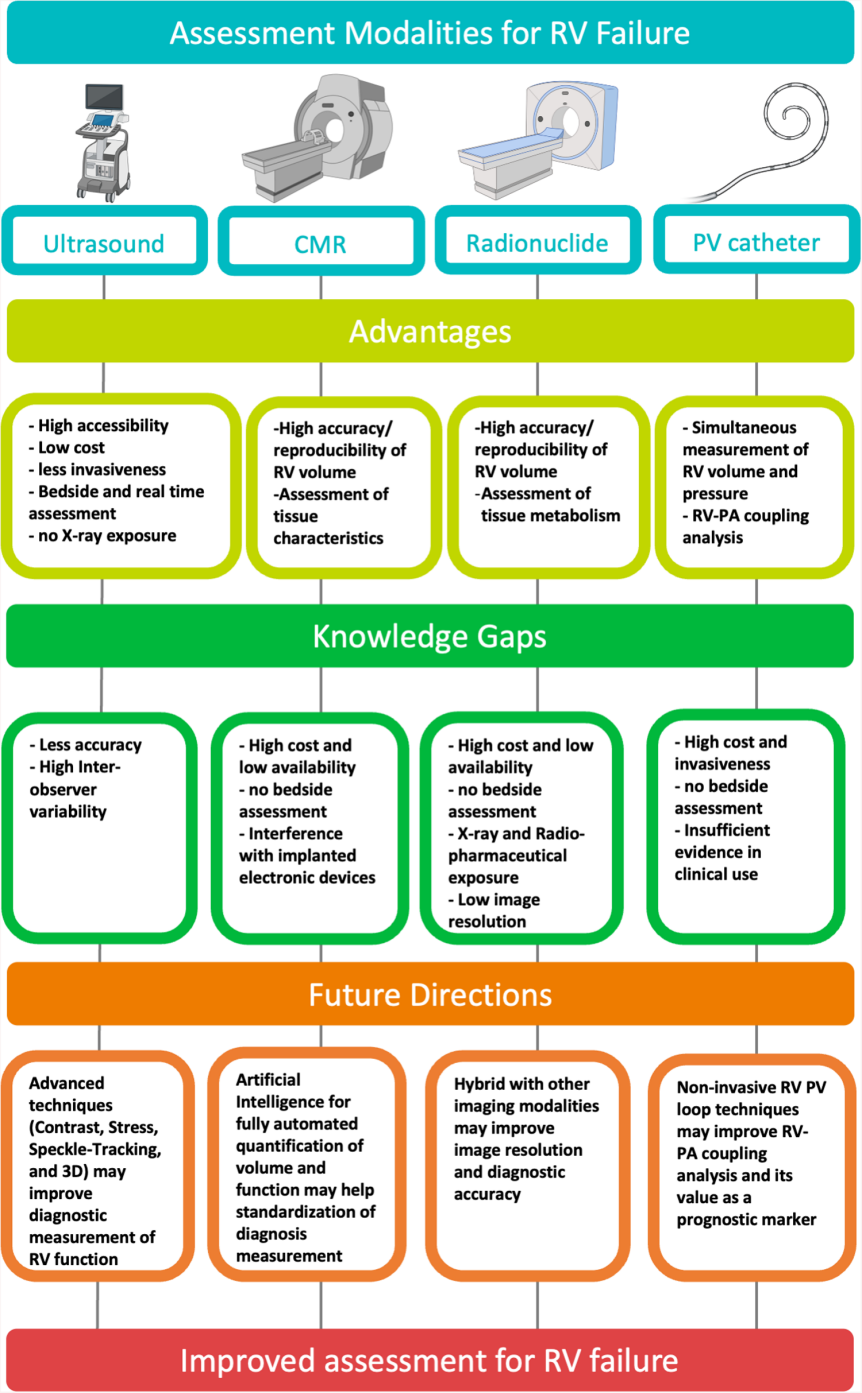
Graphical summary of the main modalities used in assessment of right ventricular failure. Created with BioRender.com. CMR, cardiac magnetic resonance; PV, pressure–volume; RV, right ventricular; PA, pulmonary artery.

There have been challenges of standardizing assessment of RV function and quantifying RVF in general, and more so in critically ill patients. Figure 4 presents a graphical summary of the main modalities used in assessment of RVF. Future modalities of RVF assessment must strive to be less invasive or non-invasive and more accessible to all patient cohorts, without compromising the accuracy or reproducibility of results.

Echocardiography, when coupled with AI analytic technology, is a promising non-invasive alternative to RV PV loop measurement. Some recent studies in recent years have begun to investigate the accuracy of AI in evaluating RVF. Zhu et al. published results recently demonstrating AI-based analysis of 3D echocardiography provided comparable estimates to CMR (150). However, a key limitation reported was that image quality was a significant determinant of analysis accuracy. Deep learning is one analytical algorithm that falls under AI and has been investigated in the context of assessing RVF. Shad *et al*. investigated prediction of RV failure in patients following implantation of a LVAD (151). The deep learning system utilized in this study to review echocardiography videos was more successful in predicting RV failure than a team of experts reviewing the same images. The utility of deep learning is not just confined to echocardiography. A 2020 study from Baskaran *et al*. demonstrated reliable identification of cardiovascular structures, including accurate reporting of RV volumes, using deep learning to analyze coronary computed tomography angiography images (151).

## 5. Conclusion

Appropriate assessment of RV function is important in predicting patients' outcome and deciding the optimal timing of treatment in various cardiac diseases. Nevertheless, standardized method for the assessment of RV function and following RVF diagnosis have not yet been established. Although imaging modalities have developed and been essential for the evaluation of RV function, more reproducible and accurate methods are required. Advanced technique such as automatic evaluation with AI and 3D assessment for the complex RV structure can play key roles for this purpose. Further noninvasive assessments for RV-PA coupling and RV-LV are also warranted to overcome the load-related limitations for the accurate evaluation of RV contractile function. Robust evidence cross-validating technologies in various etiologies is required.
